# Enhancing Human Activity Recognition through Integrated Multimodal Analysis: A Focus on RGB Imaging, Skeletal Tracking, and Pose Estimation

**DOI:** 10.3390/s24144646

**Published:** 2024-07-17

**Authors:** Sajid Ur Rehman, Aman Ullah Yasin, Ehtisham Ul Haq, Moazzam Ali, Jungsuk Kim, Asif Mehmood

**Affiliations:** 1 Department of Creative Technologies, Air University, Islamabad 44000, Pakistan; 2 Department of Biomedical Engineering, College of IT Convergence, Gachon University, 1342 Seongnamdaero, Sujeong-gu, Seongnam-si 13120, Republic of Korea; jungsuk@gachon.ac.kr; 3 Research and Development Laboratory, Cellico Company, Seongnam-si 13449, Republic of Korea

**Keywords:** Human Activity Recognition (HAR), two-stream network, skeletal extraction, 2 + 1 dimensional convolutional neural network (2 + 1D CNN), spatiotemporal feature extraction, multimodal fusion, UTD Multimodal Human Action Dataset (UTD MHAD), pose estimation, deep learning

## Abstract

Human activity recognition (HAR) is pivotal in advancing applications ranging from healthcare monitoring to interactive gaming. Traditional HAR systems, primarily relying on single data sources, face limitations in capturing the full spectrum of human activities. This study introduces a comprehensive approach to HAR by integrating two critical modalities: RGB imaging and advanced pose estimation features. Our methodology leverages the strengths of each modality to overcome the drawbacks of unimodal systems, providing a richer and more accurate representation of activities. We propose a two-stream network that processes skeletal and RGB data in parallel, enhanced by pose estimation techniques for refined feature extraction. The integration of these modalities is facilitated through advanced fusion algorithms, significantly improving recognition accuracy. Extensive experiments conducted on the UTD multimodal human action dataset (UTD MHAD) demonstrate that the proposed approach exceeds the performance of existing state-of-the-art algorithms, yielding improved outcomes. This study not only sets a new benchmark for HAR systems but also highlights the importance of feature engineering in capturing the complexity of human movements and the integration of optimal features. Our findings pave the way for more sophisticated, reliable, and applicable HAR systems in real-world scenarios.

## 1. Introduction

Human activity recognition (HAR) is increasingly critical in healthcare, security, and intelligent environments. It enables systems to interpret human actions, paving the way for adaptive and personalized technological solutions. Despite its potential, HAR faces significant challenges in accuracy, robustness, and adaptability, especially when dealing with the complexities of real-world environments. Many HAR systems utilize multimodal data, integrating inputs from various sources such as RGB imaging and skeletal data. However, the effectiveness of these systems can be hindered by the inherent constraints of individual data sources.

Addressing these challenges, our study introduces a significant approach to enhancing HAR systems by integrating both RGB imaging and skeletal data. This two-stream network leverages the unique advantages of each data type, aiming to overcome their individual limitations. The fusion of visual and skeletal information promises not only to improve the precision of activity recognition but also to increase the systems’ reliability and adaptability across varied settings.

Our study focuses on extracting a comprehensive set of features from video data using a variety of machine learning and deep learning techniques to accurately classify human activities in diverse environments.

The primary goal of this research is to validate the effectiveness of our integrated approach. Through rigorous testing on a dataset, we aim to demonstrate the benefits of combining RGB imaging with skeletal data, establishing new benchmarks for HAR system performance. Our work seeks not only to advance the technological capabilities of HAR systems but also to broaden their applicability in improving everyday life.

The integration of multiple modalities, such as RGB imaging, skeletal extraction, and advanced pose estimation features, has gained significant attention in the field of human activity recognition (HAR) in the last decade. This approach aims to enhance recognition accuracy and robustness by leveraging the strengths of each modality while compensating for their weaknesses [1]. The use of sophisticated algorithms for processing and fusing data from different modalities has shown promising results in improving activity recognition accuracy compared to systems relying on single data sources [2]. Furthermore, the scalability and adaptability of this approach to different scenarios and environments indicate its potential for broader applications in real-world settings [3]. 3D-CNNs have proven to be a powerful tool for capturing spatiotemporal features in various applications, including action recognition, medical imaging, and pose estimation. The ability of 3D-CNNs to extract features from both spatial and temporal dimensions makes them well-suited for tasks that involve analyzing volumetric or spatiotemporal data. The use of 3D Convolutional Neural Networks (3D-CNN) for action recognition has become a mainstream approach, particularly since the introduction of I3D, which has led to the proposal of many advanced 3D-CNN architectures [4] that outperform I3D in both precision and efficiency. The effectiveness of 3D-CNNs in action recognition is attributed to their ability to capture spatiotemporal features in videos, making them a natural extension of 2D-CNNs for spatial feature learning to spatiotemporal learning in videos [5]. However, it is important to note that 3D-CNNs require a large number of parameters and a huge amount of videos to learn good representation due to their complex nature.

Moreover, the use of 3D-CNNs has also been explored in the context of hand pose estimation and 3D object pose estimation. A study discussed the use of 3D-CNNs for efficient and robust hand pose estimation from single-depth images [6], while in [7] adopted transfer learning from pre-trained 2D CNNs for spatial extraction, followed by temporal encoding, before connecting to 3D convolution layers at the top of the architecture for effective spatiotemporal learning in action recognition.

The field of 3D-CNNs for skeleton-based action recognition has seen various approaches for constructing the 3D input, such as stacking pseudo-images of distance matrices or directly summing up the 3D skeletons into a cuboid. However, these methods have been found to suffer from information loss and achieve inferior performance compared to state-of-the-art techniques [8,9]. To address these limitations, a recent work proposes a significant approach of stacking heatmaps along the temporal dimension to form 3D heatmap volumes, thereby preserving all information during this process [8,9]. This innovative method aims to overcome the challenges associated with information loss in existing 3D input construction techniques. Additionally, the use of 3D-CNN instead of 2D-CNN is advocated due to its superior capability for spatiotemporal feature learning, as highlighted in previous studies [10,11]. Another study [12] focuses on preserving information through 3D heatmap volumes, highlighting the advantages of utilizing 3D-CNN for spatiotemporal feature learning in skeleton-based action recognition.

The use of graph convolutional networks (GCN) for skeleton-based action recognition has gained significant attention in recent years. GCN models human skeleton sequences as spatiotemporal graphs, allowing for the extraction of complex features from the skeletal data. One well-known baseline for GCN-based approaches is the ST-GCN, which combines spatial graph convolutions and interleaving temporal convolutions for spatiotemporal modeling [13]. Additionally, advancements in GCN-based approaches have been achieved through techniques such as adjacency powering for multiscale modeling [14] and the integration of self-attention mechanisms to enhance modeling capacity [15].

The effectiveness of GCN in skeleton-based action recognition has been demonstrated in various studies. For instance, the study [13] emphasized the significance of spatial-temporal graph convolutional networks for capturing the dynamics of human body skeletons in the context of action recognition. Furthermore, the application of GCN has extended beyond traditional action recognition to areas such as fall detection [16], two-person interaction recognition [17], and violence action recognition [18], showcasing the versatility of GCN in diverse applications.

Moreover, the limitations and challenges associated with GCN-based approaches have also been addressed in the literature. For example, the robustness and scalability of GCN have been identified as areas for improvement, highlighting the need for further research to enhance the performance of GCN in handling diverse and complex skeletal data. Additionally, the fusion of features from skeletons and other modalities has been recognized as a critical aspect that requires careful design in GCN-based approaches.

To address the challenge of skeleton-based action recognition, researchers have explored the use of convolutional neural networks (CNNs) to learn spatio-temporal representations from skeleton sequences. One approach involves modeling the skeleton sequence as a pseudo-image based on manually designed transformations, with subsequent aggregation of heatmaps along the temporal dimension into a 2D input using color encodings or learned modules [19,20]. However, this method is susceptible to information loss during the aggregation process, leading to suboptimal recognition performance. Another line of work directly converts the coordinates in a skeleton sequence to a pseudo-image with transformations, generating a 2D input of shape K × T, where K is the number of joints and T is the temporal length. Nevertheless, this input format fails to fully exploit the locality nature of convolution networks, rendering these methods less competitive than Graph Convolutional Networks (GCN) on popular benchmarks [21].

The use of two-stream networks has been proposed to design the temporal stream ConvNet, addressing issues related to large parameters and overfitting when training datasets are limited [22]. Additionally, the employment of two-stream 3D ConvNets with early fusion techniques has been demonstrated for training models from scratch using RGB and optical flow video data, indicating the versatility of this approach across different domains [23].

Furthermore, the introduction of new two-stream inflated 3D (I3D) ConvNets has enabled the learning of seamless spatiotemporal feature extractors from video, leveraging successful ImageNet architecture designs and parameters [24]. However, it is important to note that the computational cost of two-stream ConvNets is high for the requirement of optical flow, while 3D ConvNets consume significant memory due to a large number of parameters [25].

Research studies have explored various methods for multi-modal feature fusion in HAR. For instance, some approaches project multi-modal features to low-dimensional matrices and perform multiplication to capture rich interactions between the features [2]. Additionally, the use of multi-modal fusion networks has been proposed to learn multi-modal features for ground-based cloud recognition, indicating the versatility of multi-modal approaches across different domains [26]. Moreover, the combination of multi-modal features has been identified as a key factor for subsequent classifiers in the context of early-stage knee osteoarthritis disease classification [27].

Moreover, to enhance the adaptability and performance of two-stream ConvNets, an end-to-end trainable gated fusion method, namely gating ConvNet, has been proposed based on the mixture of experts (MoE) theory [28]. Additionally, multi-modal fusion networks have been employed to jointly learn ground-based cloud images and multi-modal information, with a weighted strategy for information fusion [26].

## 2. Materials and Methods

### 2.1. Dataset Description

For our study, we utilized the UTD Multimodal Human Action Dataset (UTD-MHAD) [29], which is a publicly available dataset designed for human action recognition research. The multimodal dataset consists of 27 actions performed by 4 distinct subjects. The actions include ‘sitting down’, ‘swiping left/right’, ‘throwing’, and ‘clapping’ etc. This dataset was collected and organized by the University of Texas. Each subject performed each action 4 times, and hence a total of 861 samples were collected after the removal of 3 corrupted samples.

**Figure 1:**
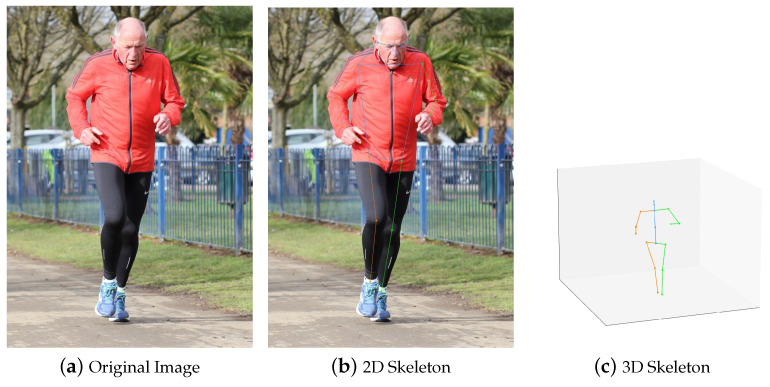
2D vs. 3D Skeleton.

The UTD-MHAD dataset is comprised of data from four different modalities: RGB videos, depth sequences, 3D skeletal data, and inertial data. An example of 2D and 3D Skeleton can be viewed in Figure 1. For the purpose of our research, we focused on the RGB videos and 2D skeletal data to leverage the visual and spatial-temporal information they provide.

The RGB videos were captured using a Microsoft Kinect camera positioned approximately 2 m in front of the subjects, ensuring a clear view of the actions being performed. The videos are recorded at a resolution of 640 × 480 pixels with a frame rate of 30 frames per second.

### 2.2. Data Preparation

**Figure 2:**
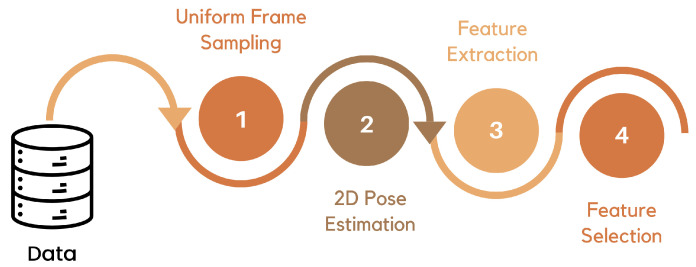
Data Preprocessing Pipeline.

This section elaborates on the crucial steps involved in preprocessing the RGB data for activity recognition using a sequence modeling approach as depicted in Figure 2. Effective data preparation is essential for extracting relevant features and ensuring optimal model performance.

#### 2.2.1. Uniform Frame Sampling

We begin by uniformly sampling a set of T frames from the video. This ensures an unbiased representation of the entire activity by selecting frames at regular intervals throughout the video duration. The sampling interval can be calculated as:

(1) $$\mathrm{Sampling}\;\mathrm{Interval}=\frac{\mathrm{Video}\;\mathrm{Duration}}{\left(T-1\right)}$$

where *T* represents the number of uniformly sampled frames from the video.

#### 2.2.2. 2D Pose Estimation

For each sampled frame, we extract the 2D pose coordinates using a pose estimation algorithm. These algorithms typically employ deep learning models trained on large datasets of labeled images containing human poses. The model analyzes the frame and identifies keypoints corresponding to different body joints (e.g., elbow, knee, shoulder). The specific keypoints extracted are listed in Table 1.

#### 2.2.3. Feature Extraction

Once the 2D pose coordinates are obtained for all T frames, we proceed to extract features relevant to activity recognition. This involves calculating:

- Distances: The Euclidean distance between body parts. The distance between keypoints is computed using the Euclidean distance formula:
  (2) $$\mathrm{distance}\left(A,B\right)=\sqrt{{\left(B_{x}-A_{x}\right)}^{2}+{\left(B_{y}-A_{y}\right)}^{2}}$$
  where ($A_{x}$, $A_{y}$) and ($B_{x}$, $B_{y}$) are the 2D coordinates of keypoints A and B, respectively.
- Angles: The relative orientation between body parts. We calculate the angles between keypoints using the arctangent function:
  (3) $$\mathrm{angle}\left(A,B\right)=\arctan\left(\frac{B_{y}-A_{y}}{B_{x}-A_{x}}\right)$$

where ($A_{x}$, $A_{y}$) and ($B_{x}$, $B_{y}$) are the 2D coordinates of keypoints A and B, respectively.

By calculating these features for each frame, we obtain a sequence of features capturing the temporal evolution of body part positions and orientations throughout the activity.

#### 2.2.4. Data Structure and Features

The features relevant to activity recognition are calculated for each frame in the video, resulting in a sequence capturing the temporal evolution of the pose. A detailed breakdown of the data structure and the number of features extracted is provided in Table 2.

#### 2.2.5. Feature Selection with Forward Feature Selection (FFS)

To enhance the efficiency and performance of our activity recognition model, we employ the forward feature selection (FFS) method as a crucial step in our data preprocessing pipeline. FFS is a technique that systematically adds features to the model one at a time, starting with the most relevant ones. The goal is to iteratively select the subset of features that optimally contributes to the model’s predictive capability.

In our case, with 307 features per frame, including frame index, angles, and distances between body parts, FFS becomes indispensable for identifying the most informative subset of features for human activity recognition. The total features encompass a comprehensive representation of the temporal evolution of body poses, but not all may be equally valuable for discerning specific activities. FFS aids in narrowing down the feature set to the most discriminative angles and distances, potentially improving model interpretability and generalization.

**Figure 3:**
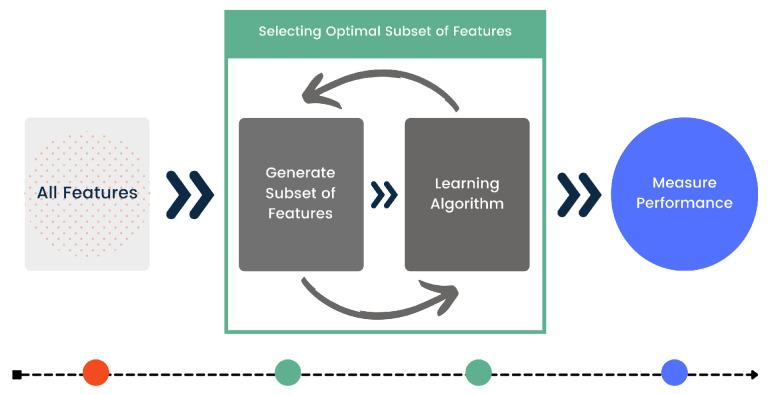
Forward Feature Selection Technique.

The FFS process involves selecting features using Random Forest before training the LSTM classification model. This approach ensures that the optimal feature subset is chosen based on its impact on model performance, as depicted in Figure 3. After the optimal features have been selected, these are used to train a more streamlined bi-directional LSTM model for better human activity recognition on the given dataset. The efficiency of this procedure may be affected by the number of features in the training data, which should be considered when scaling up. Increasing the number of features extends the selection time, but our approach maintains accuracy by consistently using the same feature subset during validation and testing phases. This consistency is critical to mitigating potential decreases in accuracy that could arise if the features in validation/testing data differ significantly from those used during training.

#### 2.2.6. Data Structure and Features (after FFS)

After applying forward feature selection, we obtain a refined set of features that optimally contribute to the activity recognition task. The updated data structure, including the selected features, will be detailed in Table 3, reflecting the improvements made through the feature selection process.

**Table 3:** Data Structure and Features (After FFS).

Component	Description	Number of Features
Frame Index	An integer value (1-indexed) indicating the order of the frame within the video.	1
2D Keypoints	A list of 19 keypoints that are selected after FFS. Refer to Table 4 for corresponding body parts.	K = 19
Selected Angles	A refined list of angles calculated between selected pairs of keypoints.	31
Selected Distances	A refined list of Euclidean distances calculated between selected pairs of keypoints.	22
Total Features	The combined number of selected angles and distances.	73

**Table 4:** Selected Keypoints After FFS.

Keypoint Coordinate
Left_eye_y
Right_eye_x
Left_ear_x
Left_ear_y
Right_ear_y
Right_shoulder_x
Left_elbow_x
Left_wrist_x
Left_wrist_y
Right_wrist_x
Left_hip_x
Right_hip_x
Right_hip_y
Left_knee_x
Left_knee_y
Right_knee_x
Right_knee_y
Left_ankle_x
Right_ankle_y

Following preprocessing, we split the UTD-MHAD dataset into training, validation, and testing sets using a common split ratio (e.g., 70% for training, 20% for validation, and 10% for testing). This split ensures the model is trained on a representative portion of the data, the validation set helps fine-tune hyperparameters, and the testing set provides an unbiased evaluation of the model’s performance on unseen data.

### 2.3. Pose Extraction

In the context of skeleton-based action recognition, the extraction of the human skeleton or pose is a critical preprocessing step that significantly impacts the final recognition accuracy. Despite its crucial role, the importance of pose extraction is often underestimated in existing literature, where poses obtained from sensors or pre-existing pose estimators are commonly utilized without thorough consideration of their potential effects. This research aims to elucidate key aspects of pose extraction to identify best practices in the field.

In the field of human pose estimation, Ref. [30] has investigated the intricacies, focusing on the correlation between 2D and 3D human pose estimation. They have studied the correlation between 2D and 3D human pose estimation, underscoring the significance of matching techniques in improving accuracy. Another piece of research that contributes to understanding the intricacies of 3D human pose cannot be disregarded [31]. They emphasize improving the effectiveness of monocular 3D pose estimation networks while considering the complexities of pose estimation tasks. Additionally, the research by [32] has examined multitask deep learning strategies for real-time 3D human pose estimation and action recognition, highlighting the advantages of integrating 2D and 3D pose estimation tasks. Synthesizing insights from the studies reveals that while 3D pose estimation provides depth information, 2D pose often demonstrates higher quality due to advancements in 2D pose estimation networks and the complexities associated with 3D pose estimation ambiguity.

**Figure 4:**
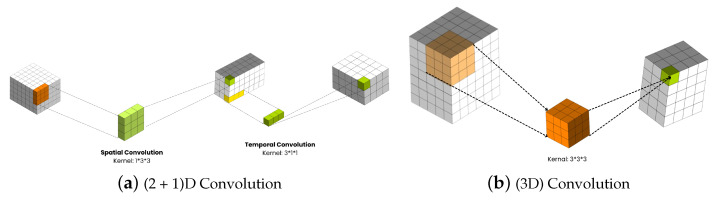
2D vs. 3D Convolution.

It is generally observed that 2D poses exhibit higher quality compared to 3D poses, as you can see in Figure 4. To extract poses, this study adopts 2D top-down pose estimators, such as those referenced in [33,34]. Top-down methods are favored over 2D bottom-up approaches due to their superior performance on established benchmarks like COCO-keypoints. The methodology involves feeding proposals generated by a human detector to the top-down pose estimators, resulting in the generation of high-quality 2D poses suitable for action recognition tasks. In scenarios where only a subset of individuals is relevant among numerous candidates, the incorporation of specific priors becomes essential for achieving optimal performance in skeleton-based action recognition.

### 2.4. 3D-CNNs

3D convolutional neural networks (3D-CNNs) have revolutionized the landscape of RGB-based action recognition by harnessing the power of both spatial and temporal information. Unlike their 2D counterparts, which analyze individual frames in isolation, 3D-CNNs process entire video sequences, capturing the inherent dynamism of actions. This shift allows them to learn motion features directly from the data, eliminating the need for laborious handcrafted features and enabling the modeling of long-range dependencies within the video.

**Figure 5:**
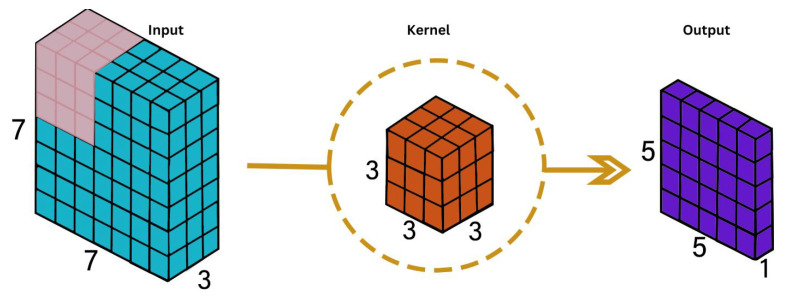
3D Convolution Operation.

This architecture generates a 3D activation map during convolution, enabling the analysis of object positions over time and in a volumetric context [35]. 3D convolution involves utilizing a three-dimensional filter to compute the representation of elements at a low level, with the kernel moving in three directions (*x*, *y*, *z*) [35] as shown in Figure 5. In the 3D convolution layer, the value at each position of the feature map is determined by the following equation:

(4) $$v_{ij}^{xyz}=\tanh\left(b_{ij}+\sum_{m}\sum_{p=0}^{P_{i}-1}\sum_{q=0}^{Q_{i}-1}\sum_{r=0}^{R_{i}-1}w_{ijm}^{pqr}x_{(i-1)m}^{\left(x+p)(y+q)(z+r\right)}\right),$$

where $w_{ijm}^{pqr}$ is the value of the kernel connected to the feature map in the previous layer, and $R_{i}$ is the size of the 3D kernel.

This convolution operation, as described in Equation (4), is crucial for extracting features in the 3D space and plays a significant role in the overall performance of the 3D convolutional neural network.

During 3D convolution, the network produces a three-dimensional volume space output by stacking adjacent layers around the center of a cube. Functional maps are interconnected to capture motion information, although each convolution kernel can only extract one type of element. The structure of a 3DCNN is similar to a 2D convolutional neural network, where combining multiple convolution layers can enhance results. The performance of a 3DCNN depends on different hyper-parameters such as the number of layers, the number of filters in each layer, the filter sizes, and the use of pooling, which in 3D data requires three values for the pooling size [35,36,37,38].

The strengths of 3DCNNs lie in their ability to learn spatiotemporal features directly from input data, making them suitable for tasks requiring the analysis of both spatial and temporal dimensions. By leveraging the capabilities of 3DCNNs, applications ranging from video classification to medical image analysis have shown significant performance enhancements [39,40]. The architecture’s capacity to extract features from spatial and temporal dimensions by convoluting in input video cubes using stereo convolutional kernels has been pivotal in various domains, including driver fatigue behavior detection and COVID-19 detection [40,41].

### 2.5. (2 + 1)D ConvNets for RGB Videos

**Figure 6:**
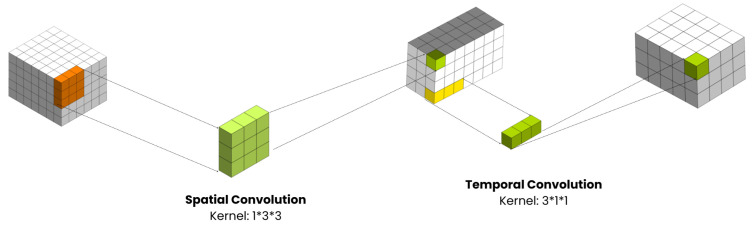
Operation to Decompose Spatial and Temporal Dimensions.

This section details the (2 + 1)D convolutional neural network model, a significant approach for processing spatiotemporal data in action recognition tasks, introduced in [8]. To enhance the spatiotemporal data processing for action recognition tasks, incorporating the (2 + 1)D convolutional neural network model proposed in [8] would be highly beneficial. This model introduces a significant approach by utilizing (2 + 1)D convolutions with residual connections, which decompose spatial and temporal dimensions as depicted in Figure 6, leading to more efficient parameter usage compared to traditional 3D convolutions. The (2 + 1)D convolutional operation involves spatial convolutions for data of shape (1, width, height) and temporal convolutions for data of shape (time, 1, 1), significantly reducing the number of parameters required for processing compared to 3D convolutions.

By implementing a (2 + 1)D ResNet18 model, where each convolutional layer in the ResNet architecture is replaced with a (2 + 1)D convolution, you can efficiently process spatiotemporal features in videos, enabling effective action recognition while minimizing the computational complexity associated with traditional 3D convolutions.

By implementing a (2 + 1)D ResNet18 model, where each convolutional layer in the ResNet architecture is replaced with a (2 + 1)D convolution, one can efficiently process spatiotemporal features in videos. This enables effective action recognition while minimizing the computational complexity associated with traditional 3D convolutions.

This adaptation allows for the capture of both spatial and temporal information in video data through a more parameter-efficient architecture, as demonstrated in the referenced paper [8].

### 2.6. Enhanced LSTM Model Architecture for Skeletal Data Processing

In addressing the complexities of human activity recognition (HAR), we have developed an enhanced long short-term memory (LSTM) architecture that introduces several key modifications to the standard LSTM model, aimed at improving the accuracy, robustness, and learning efficiency of the HAR system. The enhancements are as follows:

#### 2.6.1. Increase Model Depth

To capture more complex temporal patterns inherent in human activities, our architecture incorporates multiple LSTM layers. This depth allows the model to learn a richer hierarchy of temporal features. Careful monitoring and regularization techniques are employed to mitigate the risk of overfitting associated with increased model complexity.

#### 2.6.2. Bidirectional LSTM Layers

By incorporating bidirectional LSTM layers, our model gains the ability to process temporal data in both forward and backward directions. This bidirectionality provides a more comprehensive context for each time step, enhancing the model’s ability to understand temporal dynamics.

#### 2.6.3. Dropout for Regularization

Dropout layers are strategically placed within the network to prevent overfitting. By randomly setting a fraction (0.5 in our initial configuration) of the input units to zero at each update during training, dropout ensures that the model remains generalizable to new, unseen data.

#### 2.6.4. Batch Normalization

Following each LSTM layer, batch normalization is applied. This technique normalizes the activations and gradients, facilitating faster and more stable training. It also acts as a form of regularization, further contributing to the model’s generalizability.

#### 2.6.5. Dense Layer Configuration

The architecture concludes with dense layers, which consolidate the temporal features learned by the LSTM layers into a representation suitable for classification. The number of neurons in these dense layers is tuned to balance model complexity with performance.

#### 2.6.6. Learning Rate Scheduling

To optimize the training process, we employ a learning rate scheduling strategy that dynamically adjusts the learning rate based on the validation loss. Using the ReduceLROnPlateau callback, the learning rate is decreased when the loss plateaus, enhancing the convergence of the model.

### 2.7. Fusion Techniques for Combining Stream Probabilities

In our proposed action recognition approach, we employ a two-stream network architecture for enhanced performance. The first stream processes RGB video frames using a 2 + 1D convolutional neural network (CNN) to capture spatial and temporal features. The second stream utilizes a long short-term memory (LSTM) network to analyze pose information, including distances and angles extracted from 10 video frames. To leverage the strengths of both streams and improve prediction accuracy, we employ fusion techniques to combine the individual class probabilities predicted by each stream.

#### 2.7.1. Averaging

This method calculates the average probability across the two streams for each action class:

$$\begin{array}{cc} \mathrm{ProbFusion1(action\_class)} & =\frac{{\mathrm{Prob}}_{\mathrm{RGB}}\left(\mathrm{action\_class}\right)+{\mathrm{Prob}}_{\mathrm{Pose}}\left(\mathrm{action\_class}\right)}{2} \end{array}$$

Here, ProbRGB (action_class) denotes the probability of the action class predicted by the RGB stream, and ProbPose(action_class) represents the probability predicted by the pose stream. This approach assumes equal contribution from both streams and treats their predictions as independent.

#### 2.7.2. Multiplication

This method emphasizes agreement between the streams by multiplying the probabilities from each stream:

$$\begin{array}{cc} \mathrm{ProbFusion2(action\_class)} & ={\mathrm{Prob}}_{\mathrm{RGB}}\left(\mathrm{action\_class}\right)\cdot {\mathrm{Prob}}_{\mathrm{Pose}}\left(\mathrm{action\_class}\right) \end{array}$$

A high combined probability using multiplication indicates strong agreement between the streams for that specific action class.

#### 2.7.3. Example of Fusion Techniques

Consider an action recognition scenario where the system classifies two actions: “walking” and “jumping”. We have hypothetical probabilities obtained from the RGB and Pose streams for each action class, as shown in Table 5.

The table shows the individual stream probabilities and the combined probabilities obtained using averaging and multiplication. Averaging assigns a higher weight to the “walking” class, aligning with the higher probabilities from both streams, suggesting agreement.

In contrast, multiplication significantly reduces the combined probability for “walking” compared to averaging, indicating disagreement between the streams. The pose stream’s lower probability for “walking” outweighs the RGB stream’s high probability, leading to a lower combined score. Similarly, the very low combined probability for “jumping” using multiplication highlights strong disagreement between the streams.

This example demonstrates how different fusion techniques can lead to varying interpretations based on how they combine individual stream predictions.

### 2.8. Our Proposed Network Architecture

**Figure 7:**
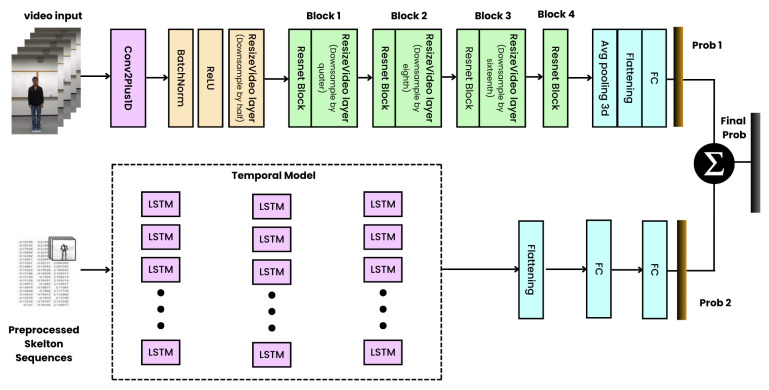
Proposed Ensemble Two Stream Deep Neural Network for HAR.

Our research introduces a state-of-the-art network architecture specifically engineered for human activity recognition (HAR). The architecture stands on the significant integration of two distinct processing streams: one dedicated to skeletal data and the other to RGB video data. This section provides a technical deep dive into each component of our architecture as shown in Figure 7, employing a two-stream approach as detailed in Algorithm 1, detailing the methodologies and technologies employed to synergistically exploit the complementary strengths of skeletal and visual information.
**Algorithm 1** Two-Stream Network for Activity Recognition with Fusion**Require:** Data:1:Skeleton data (2D pose coordinates, angles, distances)2:RGB video3:**procedure** TwoStreamActivityRecognition(skeletonData, rgbVideo)(    )4:    **Stages**5:          **Stage 1: Skeleton Stream:**6:               1. Uniformly sample 10 frames from each video of the dataset.7:               2. For each frame:8:                      a. Extract 2D pose coordinates.9:                      b. Apply Normalization on the keypoints.10:                    c. Calculate joint angles and distances.11:               3. Apply Feature Selection using FFS.12:               4. Store preprocessed data as Xs.13:               5. Feed Xs into Long Short-Term Memory (LSTM) network.14:               6. Output: Probabilities for each activity class: Prob_115:          **Stage 2: RGB Stream:**16:               1. Feed RGB video into a 2 + 1D Convolutional Neural Network (CNN).17:                  - Utilize 2D spatial convolutions for feature extraction.18:                  - Utilize 1D temporal convolution for capturing temporal dependencies.19:               2. Output: Probabilities for each activity class: Prob_220:          **Stage 3: Fusion:**21:               - Perform fusion using chosen methods (e.g., addition, multiplication):22:                  a. Prob_Fusion1 = Prob_1 + Prob_2 (Addition)23:                  b. Prob_Fusion2 = Prob_1 × Prob_2 (Multiplication)24:          **Stage 4: Decision:**25:               - Choose the prediction with higher confidence:26:                  a. Pred_Fusion1 = max(Prob_Fusion1)27:                  b. Pred_Fusion2 = max(Prob_Fusion2)28:**end procedure**

#### 2.8.1. Conceptual Framework of the Two-Stream Approach

At the heart of our architecture lies a dual-pathway strategy purposefully designed to independently process and extract features from skeletal and RGB video data. This bifurcated approach is not merely parallel processing but a deliberate effort to capture the unique attributes each modality offers—the dynamism of human movement through skeletal data and the contextual richness through visual data. A sophisticated fusion mechanism at the architecture’s terminus integrates the learned features, providing a holistic insight into the activities being recognized.

#### 2.8.2. Enhanced LSTM for Skeletal Stream Processing

The skeletal data stream is powered by an enhanced long short-term memory (LSTM) model, which includes several strategic enhancements to the conventional LSTM architecture, making it particularly proficient at capturing the temporal sequences and intricacies of human motion:

- Data Preprocessing and Normalization: A meticulous preprocessing pipeline standardizes the skeletal data, involving frame selection based on uniform temporal intervals, normalization of pose coordinates to a consistent scale, and the derivation of spatial features like joint angles and inter-joint distances, thereby enriching the input feature set.
- Advanced Feature Selection: Employing a sophisticated feature-based feature selection (FFS) algorithm, our model efficiently identifies and retains the most predictive features from the skeletal data, significantly reducing dimensionality while preserving critical temporal and spatial information essential for accurate activity recognition.
- Architectural Enhancements in LSTM:
  – Bidirectional Processing: Incorporating bidirectional LSTM layers enables the model to learn from both past and future contexts, offering a more comprehensive understanding of temporal sequences.
  – Regularization Techniques: Dropout layers are integrated throughout the network as a regularization measure to combat overfitting, alongside batch normalization to facilitate faster and more stable convergence.
  – Optimized Dense Layers: The architecture concludes with carefully configured dense layers that act to consolidate and interpret the learned temporal features, fine-tuned for optimal classification performance.

#### 2.8.3. 2 + 1D CNN for RGB Video Stream Processing

Concurrently, the RGB video data stream employs a 2 + 1D Convolutional Neural Network (CNN), a cutting-edge model tailored for extracting spatiotemporal features:

- Spatiotemporal Decomposition: The 2 + 1D CNN uniquely separates the convolution operations into spatial (2D) convolutions for extracting spatial features from individual video frames, capturing the visual appearance of the activity, and temporal (1D) convolutions to capture temporal dependencies across video frames, understanding how the activity unfolds visually over time, thereby efficiently learning from the RGB data.
- Extraction and Learning: This innovative architecture facilitates the precise extraction of visual features and their temporal evolution, which is crucial for recognizing activities from the visual cues and contexts provided in the video data.

#### 2.8.4. Fusion Strategy for Enhanced Decision Making

The integration of insights from both streams is achieved through an advanced fusion strategy, which meticulously combines the probabilistic outputs from the skeletal and RGB streams. This process uses sophisticated algorithms to ensure that decision-making leverages the full spectrum of learned features, leading to more accurate and robust activity recognition:

- Fusion Techniques: Various techniques, including weighted averaging and feature concatenation, are explored to find the optimal method for combining outputs, aiming to maximize the complementary information from both modalities.
- Confidence-Based Classification: The fused data then undergo a final classification step, where the activity with the highest cumulative confidence score is selected as the recognized activity, ensuring that the decision reflects a holistic analysis of both skeletal and visual data.

By employing this two-stream network architecture with fusion, the model effectively utilizes both skeletal and visual information, potentially leading to improved performance compared to single-modality approaches in recognizing human activities.

#### 2.8.5. Implementation Details

Our network is implemented using the TensorFlow and Keras frameworks, with a focus on optimization for performance and efficiency. Training incorporates advanced techniques such as EarlyStopping and adaptive learning rate adjustments (ReduceLROnPlateau) to fine-tune the model’s learning process, ensuring peak accuracy without overfitting.

This detailed exposition of the proposed dual-stream network architecture demonstrates its effectiveness in combining and analyzing both skeletal and RGB video data. By integrating these modalities, the architecture not only achieves a new benchmark for accuracy and reliability in human activity recognition but also paves the way for transformative advancements in various fields, for example, healthcare, sports analytics, and beyond.

## 3. Experiment and Results

This section provides the experimental environment and results. The results are evaluated and discussed at the end of this section.

### 3.1. Experimental Setup

In Table 6, a list of essential software is depicted that was used to setup the experimental environment. All experiments are performed on the Ubuntu 20.04 operating system. The Anaconda version is used to create virtual environments. Several software libraries mentioned in the table are used for loading, preprocessing, and visualizing the data. The major libraries used in this experimentation are TensorFlow-GPU, CV2, Matpplotlib, and the Python Image Library (PIL).

### 3.2. Training

The training, validation, and test data were split into 70%, 20%, and 10%, respectively. The validation data are performed along with training to evaluate the model to reduce the chance of overfitting. Training details for CNN and LSTM are shown in Table 7.

### 3.3. Results Analysis and Performance

**Figure 8:**
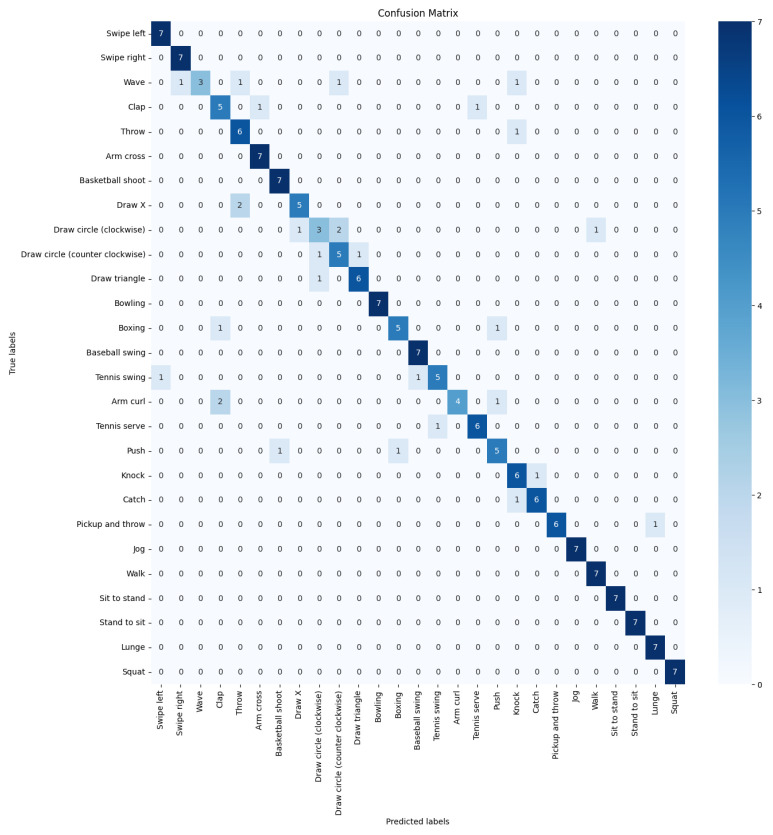
Confusion Matrix for the Enhanced LSTM Model.

In this study, we have developed and evaluated a two-stream network architecture integrating skeletal data processed by an enhanced long short-term memory (LSTM) network with RGB video data processed using a (2 + 1)D convolutional neural network (CNN). This approach leverages the unique strengths of both data types through sophisticated fusion techniques to enhance recognition accuracy significantly. (See Figure 8).

#### Performance of the Enhanced LSTM for Skeletal Data

The enhanced LSTM model is specifically designed to process skeletal data. It utilizes bidirectional layers and advanced regularization techniques, enabling it to effectively capture the dynamic temporal patterns of human activities. This section presents a comprehensive overview of the model’s performance, evaluated through both quantitative metrics and classification accuracy visualizations. The model’s performance is quantified in terms of test loss, accuracy, precision, and recall, as detailed in the Table 8.

**Figure 9:**
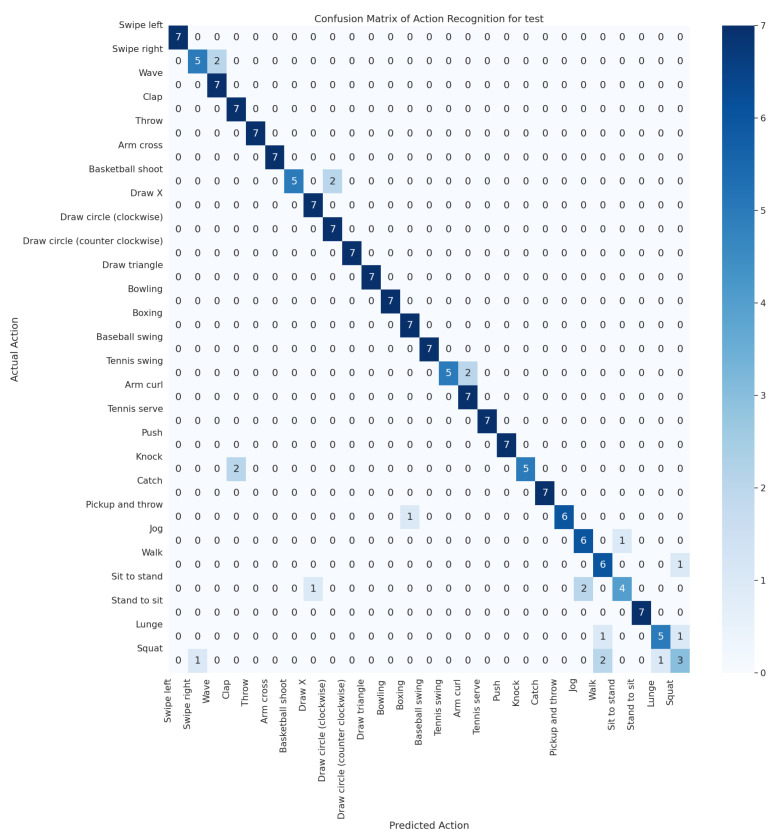
Confusion Matrix for the (2 + 1)D CNN Model.

The confusion matrix (Figure 9) provides a detailed view of the classification accuracy across different activity categories within the UTD-MHAD dataset. This visual helps identify how well the model discriminates between various types of activities.

The matrix reveals high precision in recognizing actions such as `walking’ and `sitting’, but it indicates some confusion between similar activities like `Draw Circle (clockwise)’ and ‘Draw Circle (counter clockwise)’. These insights suggest areas where the model could be further refined to reduce incorrect classifications. The performance metrics of this network on the test dataset are shown.

**Figure 10:**
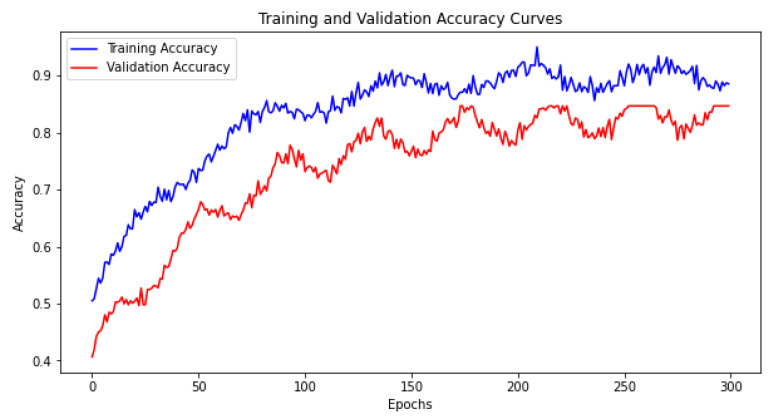
Training and validation accuracy curves for the Enhanced LSTM Model.

The model’s training process is further illustrated by the accuracy curves over 300 epochs, as shown in Figure 10. These curves demonstrate the model’s ability to learn effectively over time without overfitting. The graph shows a steady increase in training and validation accuracy, confirming the model’s good generalization capabilities across epochs.

### 3.4. Performance of the (2 + 1)D CNN for RGB Video Data

**Figure 11:**
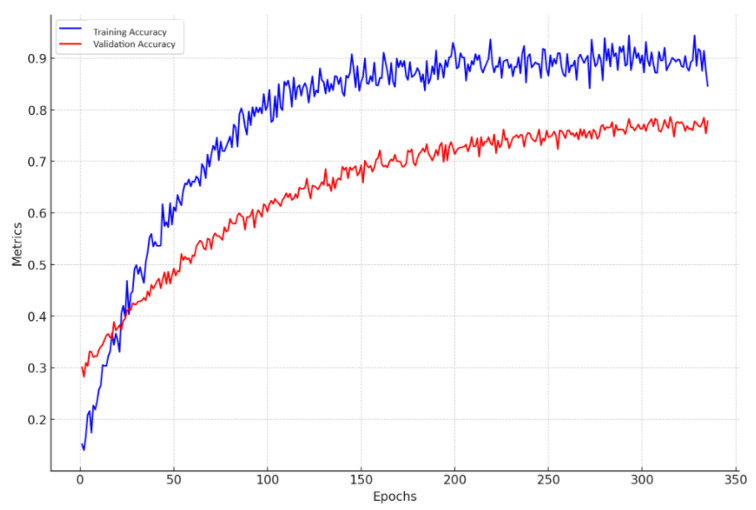
Accuracy Curves for the (2 + 1)D CNN Model.

The (2 + 1)D CNN model is strategically engineered to process RGB video data, making it proficient at efficiently extracting spatiotemporal features. The performance of this innovative model is quantitatively represented through accuracy curves over a span of 335 epochs, as shown in Figure 11. These curves display the training and validation accuracy of the (2 + 1)D CNN model over 335 epochs, underscoring the model’s learning stability and efficiency.

The accuracy curves depict a consistent upward trajectory in both training and validation accuracies, which indicates the model’s capability to generalize well across the dataset without significant signs of overfitting. The steady rise in accuracy, along with a slight divergence at later epochs, suggests potential areas for refining training parameters, such as making adjustments to the learning rate or implementing early stopping to prevent overfitting.

Additionally, the effectiveness of this model in distinguishing between different activities is further validated through its confusion matrix, as depicted in Figure 9.

This confusion matrix provides a detailed view of the classification accuracy across different activity categories, highlighting areas where the model excels and where improvements could be beneficial.

The confusion matrix reveals detailed insights into the classification performance across various activities. While the model shows high accuracy for most activities, there are notable incorrect classifications that suggest areas for further model tuning. For instance, activities with similar motion patterns may be confused, indicating a need for more nuanced feature extraction capabilities or an enhanced training dataset to better differentiate between such activities.

These results collectively demonstrate the robustness of the (2 + 1)D CNN model in handling complex video data, making it a valuable component of our two-stream network architecture for human activity recognition. The performance metrics for this stream are detailed in Table 9.

### 3.5. Fusion Results

We employed fusion techniques to combine the predictive outputs from both skeletal and RGB video data streams to leverage the complementary information from both sources. This fusion approach significantly improved the overall accuracy of human activity recognition. The resulting accuracy achieved through the fusion of both streams is presented in Table 10, along with precision, recall, and F1 Score.

### 3.6. Comparative Analysis with State-of-the-Art Models

Our proposed two-stream architecture with fusion achieves a remarkable accuracy of 98.94%, surpassing existing state-of-the-art (SOTA) models on the dataset. Table 11 offers a detailed comparison of our approach with other prominent methods.

## 4. Discussion

Our approach to human activity recognition (HAR) introduces several technical innovations that significantly contribute to the enhanced performance of our two-stream network architecture. These innovations address the inherent complexities of HAR tasks, especially the need for effective temporal sequence modeling and the integration of multimodal data for improved accuracy. Here, we delve deeper into the technical aspects that underpin our methodology.

### 4.1. Advanced Temporal Modeling with Enhanced LSTM

The enhanced LSTM architecture represents a substantial technical advancement over traditional LSTM models, particularly in its ability to model complex temporal sequences inherent in human activities. Key technical enhancements include:

#### Bidirectional Processing

Utilizing bidirectional LSTMs allows the model to capture temporal dependencies both forward and backward in time. This is crucial for understanding the full context of a sequence, as certain activity patterns may be better recognized when considering both preceding and following actions.

### 4.2. Layer-Wise Regularization and Normalization

The incorporation of dropout and batch normalization at strategic points in the architecture serves a dual purpose. Dropout acts as a form of regularization by randomly “dropping” a subset of activations, thus preventing the model from relying too heavily on any single feature and encouraging the development of more robust feature representations. Batch normalization standardizes the inputs to a layer, reducing internal covariate shift and allowing for higher learning rates and more stable training dynamics. Together, these techniques enhance the model’s generalization capability.

#### Depth and Complexity

The increase in model depth through additional LSTM layers allows for the capture of more abstract and complex temporal patterns. This depth is carefully balanced with the model’s capacity and the risk of overfitting through the aforementioned regularization strategies.

### 4.3. Spatiotemporal Feature Extraction with (2 + 1)D CNN

The (2 + 1)D CNN model is a technical innovation designed to efficiently process spatiotemporal data. This model disentangles the spatial and temporal components of convolution, offering a nuanced approach to video analysis:

#### 4.3.1. Decomposed Convolutional Operations

By separating 3D convolutions into spatial (2D) and temporal (1D) components, the model efficiently captures the distinct aspects of spatial appearances and temporal evolution. This decomposition not only reduces the computational complexity but also enhances the model’s ability to learn discriminative features for HAR.

#### 4.3.2. Optimized Frame Sampling

The technique of uniform frame sampling addresses the challenge of temporal variability across video sequences. By selecting a fixed number of frames at uniform intervals, the model ensures a representative temporal coverage of the activity, enhancing the reliability of the extracted features.

### 4.4. Fusion Strategies for Improved Predictive Performance

The fusion of skeletal and RGB video data streams is a critical component of our approach, enabling the integration of complementary information from different modalities:

#### 4.4.1. Weighted Fusion Techniques

Beyond simple averaging and multiplication, exploring weighted fusion strategies allows for a nuanced combination of predictive outputs, where the contribution of each stream can be adjusted based on its reliability or relevance to specific activities.

#### 4.4.2. Confidence and Context-Aware Fusion

Leveraging the confidence scores of predictions from each stream enables a more context-aware fusion strategy. For instance, if one stream exhibits high confidence in its prediction while the other does not, the fusion mechanism can prioritize the more confident prediction, potentially leading to more accurate overall outcomes.

#### 4.4.3. Limitations and Future Work

While the proposed approach produces promising results, it is important to note that there are some limitations associated with the size and inclusiveness of the validation dataset. With 24 videos per action, the model faces challenges in generalizing well to the validation set. We acknowledge this limitation and are exploring methods to mitigate such fluctuations in future studies.

### 4.5. Summary and Future Directions

The technical enhancements detailed in our enhanced LSTM and (2 + 1)D CNN models, coupled with our advanced fusion strategies, underpin the success of our two-stream network architecture in HAR tasks. These innovations address key challenges in HAR, such as the effective modeling of temporal sequences, the efficient processing of spatiotemporal video data, and the integration of multimodal information.

Future research directions may include the exploration of more sophisticated fusion algorithms that dynamically adjust based on context or activity type, the integration of additional data modalities (e.g., depth information or audio signals), and the application of transfer learning and domain adaptation techniques to enhance the model’s performance across diverse datasets and real-world scenarios.

## 5. Conclusions

In conclusion, our study successfully demonstrates the efficacy of a significant two-stream network architecture that integrates skeletal data and RGB video streams for the purpose of human activity recognition (HAR). The unique approach of utilizing an enhanced long short-term memory (LSTM) model to process skeletal data, alongside a (2 + 1)D convolutional neural network (CNN) for RGB video data, represents a significant advancement in the field. Our method’s superiority lies in its ability to capture and analyze the complementary information offered by both modalities, which is further enhanced through innovative fusion techniques.

The experimental results underscore the robustness and accuracy of our model, showcasing notable improvements in activity recognition performance when compared to existing methodologies. The fusion strategies employed not only amplify the strengths of each data stream but also mitigate their individual limitations, leading to a highly reliable recognition system. This research not only sets new performance benchmarks on the UTD MHAD but also highlights the critical role of integrated multimodal analysis in understanding complex human activities.

Furthermore, the exploration of enhanced LSTM features, such as bidirectional processing and advanced regularization techniques, contributes to the model’s effectiveness in capturing complex temporal dynamics. Simultaneously, the (2 + 1)D CNN approach facilitates an efficient extraction of spatiotemporal features from video data, ensuring a comprehensive understanding of the activities being performed.

Looking ahead, the potential applications of our research are vast and varied, spanning from healthcare and surveillance to interactive gaming and beyond. The adaptability and scalability of our proposed model hold promise for further research and development, aiming to explore additional data modalities and fusion techniques. Ultimately, our work lays the foundation for more sophisticated, accurate, and versatile HAR systems, moving the field one step closer to realizing its full potential in improving human-computer interaction and understanding human behavior in an array of contexts.

## Figures and Tables

**Table 1:** Keypoints Extracted by mmpose (COCO Format).

Keypoint Name	Index
Nose	0
Left eye	1
Right eye	2
Left ear	3
Right ear	4
Left shoulder	5
Right shoulder	6
Left elbow	7
Right elbow	8
Left wrist	9
Right wrist	10
Left hip	11
Right hip	12
Left knee	13
Right knee	14
Left ankle	15
Right ankle	16

**Table 2:** Data Structure and Features (Before FFS).

Component	Description	Number of Features
Frame Index	An integer value (1-indexed) indicating the order of the frame within the video.	1
2D Keypoints	A list of 17 keypoint coordinates, where each keypoint is represented by a tuple of (x, y) coordinates. Refer to Table 1 for corresponding body parts.	K = 17 (x, y coordinates)
Angles	A list of angles calculated between all pairs of keypoints.	K × (K − 1)/2 = 17 × (17 − 1)/2 = 136
Distances	A list of Euclidean distances calculated between all pairs of keypoints.	K × (K − 1)/2 = 17 × (17 − 1)/2 = 136
Total Features	The combined number of coordinates, number of angles, and distances calculated from all keypoint pairs.	306

**Table 5:** Example of Fusion Techniques for Action Recognition.

Action Class	RGB Stream Probability	Pose Stream Probability	Combined Probability	
			**Averaging**	**Multiplication**
Walking	0.7	0.8	$0.75=\frac{0.7\;+\;0.8}{2}$	0.56=0.7×0.8
Jumping	0.3	0.2	$0.25=\frac{0.3\;+\;0.2}{2}$	0.06=0.3×0.2

**Table 6:** Experimental Setup.

Name	Detail
Operating System (OS)	Ubuntu 20.04
Conda	22.9.0
python	3.10.3
jupyter notebook	6.5.2
tensorflow-gpu	2.9.0
cv2	4.9.8
matplotlib	3.8.3
pandas	2.2.1
numpy	1.26.4
PIL	10.2.0

**Table 7:** Training configurations.

Metric	Value	Metric	Value
number of epochs for CNN	355	initial learning-rate	0.001
number of epochs for LSTM	300	batch-size	16
optimizer	Stochastic Gradient Descent (SGD)		

**Table 8:** Performance Metrics of Enhanced LSTM for Skeletal Data.

Metric	Validation Loss	Validation Accuracy	Precision	Recall
Value	0.4970	84.66%	85.53%	84.66%

**Table 9:** Performance Metrics of (2 + 1)D CNN for RGB Video Data.

Metric	Test Loss	Test Accuracy	Precision	Recall
Value	0.2928	89.42%	86.49%	89.94%

**Table 10:** Fusion Results.

Fusion Method	Accuracy	Precision	Recall	F1 Score
Averaging	96.30%	96.97%	96.30%	96.32%
Multiplication	98.94%	99.00%	98.94%	98.93%

**Table 11:** Comparison with State-of-the-Art Models on the Human Activity Recognition Dataset.

Model	Accuracy
Action Machine: Rethinking Action Recognition in Trimmed Videos [42]	92.8%
STAR-Net: Action Recognition using Spatio-Temporal Activation Reprojection [43]	90.0%
A Closer Look at Spatiotemporal Convolutions for Action Recognition [8]	89.64%
Ours (Enhanced LSTM + (2 + 1)D CNN) Fusion	98.94%

## Data Availability

The original contributions presented in the study are included in the article, further inquiries can be directed to the corresponding author.
